# Supplementary material for: Perioperative Geriatric Assessment as A Predictor of Long-Term Hepatectomy Outcomes in Elderly Patients with Hepatocellular Carcinoma
Source: Cancers (Basel). 2021 Feb 17;13(4):842. doi: 10.3390/cancers13040842 (PMC7922697; doi:10.3390/cancers13040842)
Supplement: Supplementary file 1 [file cancers-13-00842-s001.pdf]

Supplementary Materials: Perioperative Geriatric Assessment as A Predictor of Long-Term Hepatectomy Outcomes in Elderly Patients with Hepatocellular Carcinoma

Masaki Kaibori, Hideyuki Matsushima, Morihiko Ishizaki, Hisashi Kosaka, Kosuke Matsui, Asao Ogawa, Kengo Yoshii and Mitsugu Sekimoto

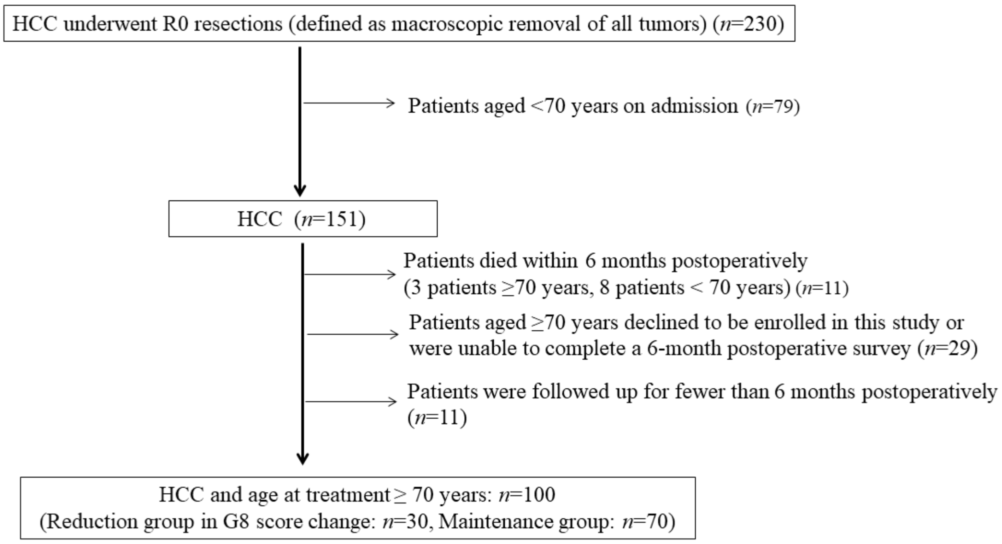

Figure S1. Patient flow chart.
